# Truthful Channel Sharing for Self Coexistence of Overlapping Medical Body Area Networks

**DOI:** 10.1371/journal.pone.0148376

**Published:** 2016-02-04

**Authors:** Gengfa Fang, Mehmet A. Orgun, Rajan Shankaran, Eryk Dutkiewicz, Guanglou Zheng

**Affiliations:** 1 Department of Engineering, Macquarie University, Sydney, NSW 2109, Australia; 2 Department of Computing, Macquarie University, Sydney, NSW 2109, Australia; 3 School of Computing and Communications, University of Technology, Sydney, Broadway, NSW 2007, Australia; Tianjin University of Technology, CHINA

## Abstract

As defined by IEEE 802.15.6 standard, channel sharing is a potential method to coordinate inter-network interference among Medical Body Area Networks (MBANs) that are close to one another. However, channel sharing opens up new vulnerabilities as selfish MBANs may manipulate their online channel requests to gain unfair advantage over others. In this paper, we address this issue by proposing a truthful online channel sharing algorithm and a companion protocol that allocates channel efficiently and truthfully by punishing MBANs for misreporting their channel request parameters such as time, duration and bid for the channel. We first present an online channel sharing scheme for unit-length channel requests and prove that it is truthful. We then generalize our model to settings with variable-length channel requests, where we propose a critical value based channel pricing and preemption scheme. A bid adjustment procedure prevents unbeneficial preemption by artificially raising the ongoing winner’s bid controlled by a *penalty factor* *λ*. Our scheme can efficiently detect selfish behaviors by monitoring a *trust* parameter *α* of each MBAN and punish MBANs from cheating by suspending their requests. Our extensive simulation results show our scheme can achieve a total profit that is more than 85% of the offline optimum method in the typical MBAN settings.

## Introduction

Medical Body Area Networks (MBANs) are key technology to facilitate ubiquitous healthcare services for patient management such as remote patient monitoring, disability management and remote control of wireless devices. MBANs can be used to monitor vital body signs such as heart rate, temperature, blood pressure, electrocardiogram (ECG), electroencephalogram (EEG) and pH level of patients. By replacing cables with wireless links, MBANs can provide less invasive and more comfortable ways to monitor a patient, both in and outside the hospital environment and are efficient since MBANs pose minimal infection risks.

Unlike cellular networks, MBANs are randomly distributed networks where two or more of them may deeply overlap and interfere with each other due to the limited available frequency bands. For example in a crowded bus more than 10 people could sit close to each other, such that each person’s MBAN may severely interfere with the other MBANs in the vicinity. Such severe interference will decrease the Signal to Interference and Noise Ratio (SINR) potentially risking patients’ lives.

To mitigate the severe inter-network interference among overlapping MBANs, IEEE 802.15.6 standard [1] proposed several interference mitigation schemes such as beacon shifting, channel hopping and channel sharing (i.e., active superframe interleaving), but has not described the underlying details that relate to a specific protocol and algorithm to achieve low or no interference. In this paper, we explore the problem of channel sharing for the self coexistence of multiple and deeply overlapping MBANs.

Channel sharing among MBANs introduces significant design challenges because of the mobility and safety requirements of MBANs. First, the channel sharing scheme must be on demand, so that MBANs can request channel access frame by frame. Second, unlike the previous studies on channel assignment (e.g., [2, 3]), the channel access scheme has to make decisions on-the-fly, i.e., without the knowledge of future events such as which networks will subsequently join or leave the channel sharing spectrum. Third, channel sharing must be self enforcing so that each MBAN will benefit from sharing the channel with other MBANs instead of operating uncooperatively and causing interference to others. Finally, the channel sharing scheme must enforce truthfulness so that no single MBAN can gain unfair advantage by manipulating its channel request parameters and thereby denying access to other legitimate MBANs.

In this paper, we model the channel sharing problem as an online channel access auction, wherein the participating users (i.e., MBANs) will request channel access whenever channel bandwidth is needed to support the communications in the networks. According to the online auction theory [4, 5], each request consists of a monetary bid and channel access information which includes the start time, end time and channel access length that could be utilized in the channel sharing problem. One user in the network takes the role of an auctioneer. The auctioneer processes the requests on-the-fly, and forwards appropriate channel access *grant* messages to all the users. By decoding the channel access grants, each user can access the channel that is granted to him. In this way, users can request and share the channel with their neighbours thereby avoiding collisions which may lead to severe inter-network interference.

Although the online auction based channel access scheme seems to address the problem on inter MBAN interference in an efficient way, it opens up vulnerabilities for selfish users who could try to gain an unfair advantage over others by manipulating their request parameters. For example, a user can falsely report its channel request in terms of the channel access duration, start time and end time, or bid to get more channel bandwidth at a lower cost. Manipulation of channel access requests can result in the blocking of other users from transmitting their packets. Therefore, an efficient online channel access auction design for MBANs’ coexistence needs to address this critical problem so that misbehaving users will be punished and as a result, channel bandwidth can be fairly shared among users.

To discourage users from manipulating their channel access requests, we propose an online truthful channel sharing scheme that maximizes users’ profit if and only if users truthfully report their channel requests. To the best of our knowledge, this is the first study to address both the network coexistence issue of MBANs (defined in IEEE 802.15.6 standard) as well as truthful channel sharing problem simultaneously.

Our proposed scheme combines a monotone channel allocation rule with a payment mechanism. The monotone channel allocation rule allocates the channel to the user with the highest bid who has so far not been allocated the channel in the current frame. Apart from the channel allocation rule, we propose a payment mechanism that computes the payment for each successful bidder of each frame. The central idea with payments is that a user that is allocated a channel pays the smallest satisfaction value it could have reported and still received an allocation. We prove that the proposed channel sharing scheme is truthful and can achieve the overall high competitive ratio and profit.

The main contributions of this paper are summarized as follows:

- We analyse the truthful practical channel sharing problem in MBANs. We find that the channel allocation monotonicity is necessary for achieving truthfulness and also show that payment must be bid-independent and satisfy monotonicity requirements with respect to the start-time and end-time, channel access length and bid.
- Driven by the monotonic allocation methodology, we design efficient online channel sharing schemes for both unit-length and variable-length request cases. We apply a *trust* factor *α* to monitor the selfish behaviour of users and a *penalty factor* λ based bid adjustment to prevent unbeneficial preemption thereby maximizing the overall profit. We prove that our proposed methods are indeed truthful.
- We design a practical channel sharing protocol for IEEE 802.15.6 MBANs based on the truthful channel sharing schemes proposed above. In the protocol, we define a self CO-existence Period (COPE) based frame structure together with the request and grant schemes to facilitate channel sharing among multiple MBANs.
- Finally, we conduct extensive simulation based experiments to analyze the performance of the proposed truthful channel sharing algorithm and the companion protocol to show that our proposed scheme maintains fairness while achieving a total profit that is more than 85% of the offline optimum.

The rest of the paper is organized as follows. The Methods section first defines the channel sharing problem for the coexistence for multiple overlapping MBANs. We then propose a channel sharing scheme that is truthful along with a channel allocation and payment rule set to solve the channel sharing problem. A practical channel sharing protocol is developed based on the IEEE 802.15.6 standard. In the Results Section, the performance of the protocol is analyzed using simulation based experiments. In the Discussion section, we contrast our approach to the channel sharing problem with the existing works and conclude with a brief discussion of future work.

## Methods

### Channel Sharing Problem Model

We consider *n* users, i.e., MBANs sharing a unique channel frame by frame, and each frame is identical for all the users. A central authority, for example, one of the MBANs together with other management entities, decides the allocation of each frame to *n* users. The decision and nomination of the central authority is outside the scope of the paper. In other words, we assume that each user will trust the central authority and is satisfied by the outcome of the auction. Upon detecting a need for channel bandwidth from the application layer of a node, users send requests to the central authority to access the channel for a specific time period. Each request of the user *i* is characterized by a tuple *v*_*i*_ = (*a*_*i*_, *d*_*i*_, *l*_*i*_, *w*_*i*_). We refer to *a*_*i*_ and *d*_*i*_ as the request start time and end time respectively, and refer to *l*_*i*_ and *w*_*i*_ as channel length and bid accordingly. Time is slotted frame by frame. In our design, we require that the maximum channel length of each request is Δ frames. All the requests arrive in the channel sharing window and have a channel length between [1, Δ], i.e., *l*_*i*_ ∈ [1, Δ] indicating the number of frames required. We have *a*_*i*_ ≤ *d*_*i*_, 0 < *l*_*i*_ ≤ (*d*_*i*_ − *a*_*i*_) and 0 ≤ *w*_*i*_ < ∞. Each user can participate in the channel sharing procedure by simply sending its request *v*_*i*_ to the central authority. Upon receiving the requests, the central authority allocates each user a subset of the frames so that users are assigned disjoint subsets, during which the communication within that MBAN can take place according to the IEEE 802.15.6 standard for MBANs. In this paper, we assume a simple scenario where there is only one channel available. We leave it as a future work to extend the proposed channel sharing and auction schemes to the case where multiple channels are available.

In this paper, we model the above channel sharing problem as an online auction where each user (bidder) submits requests *v*_*i*_ to the auctioneer (central authority) which allocates the underlying channel to users. The online auction procedure is triggered by the arrival of a new request or the departure of the current winner. The online channel sharing scheme needs to make decisions on-the-fly, i.e., without the knowledge of oncoming future requests. As a result, the overall performance of online channel sharing can be largely degraded if users deliberately try to manipulate their requests. We use *v* = (*v*_1_, *v*_2_, …, *v*_*n*_) to denote the requests from *n* users. The auction based online channel sharing scheme includes a channel allocation rule *q* and a payment rule *p*, where *q*(*v*) = (*q*_1_, *q*_2_, …, *q*_*n*_) represents the channel allocation result, and *p*(*v*) = (*p*_1_, *p*_2_, …, *p*_*n*_) represents the amount user *i* needs to pay. Since we consider one channel in this paper, we have $\sum_{i}^{n}a_{i}=1$. We will assume that users have a quasi-linear profit function, so the profit of user *i* for allocation *q*_*i*_(*v*) and *p*_*i*_(*v*) is *q*_*i*_(*v*)*w*_*i*_ − *p*_*i*_(*v*).

To evaluate the performance of an online algorithm, we usually compare its performance with that of an offline optimal algorithm. We define the performance of the online channel sharing problem A as the competitive ratio $r(A)=min_{s}\frac{A(s)}{OPT(s)}$, where *s* represents any possible sequence of requests. *OPT*(*s*) is the profit produced by the optimum offline method and A(s) is the profit produced by the online channel sharing scheme. Our design goal is to maximize the competitive ratio while maintaining the truthfulness, i.e., malicious users can’t improve their profits.

The flexibility of the online auction method makes the channel sharing design procedure difficult and challenging. Selfish and malicious users can utilize this flexibility to manipulate their requests to control the auction outcome so as to gain an unfair advantage over others. In online channel sharing, users can cheat by not only rigging their channel length and their bids for accessing the channel, but also by falsely reporting their start and end times for accessing the channel. A good online channel access design needs to be resistant to these selfish and malicious behaviors. One well-known solution is to make the online auction truthful, i.e., no user can improve its profit by misreporting the request.

It has been shown that it is impossible to achieve a bounded competitive ratio on the efficiency of the truthful scheme without any restriction being imposed upon the types of misreporting [6]. In this paper, we assume that users are selfish; as a result a user will not report channel request that has a start-time earlier than its true start-time and an end-time later than its true end-time. This is a practical and reasonable assumption for MBAN channel sharing problem, because each user will not benefit if it reports the channel usage time to be earlier than it really needs it. In addition, delaying its channel usage will be disadvantageous to the user. Since each user is selfish, it will only misreport its channel request with no earlier start-time and no later end-time.

In this paper, we propose a truthful online channel scheme. If we use $v_{i}^{{}^{\prime }}=\left(a_{i}^{{}^{\prime }},d_{i}^{{}^{\prime }},l_{i}^{{}^{\prime }},w_{i}^{{}^{\prime }}\right)$ to denote the selfish request and *v*_*i*_ = (*a*_*i*_, *d*_*i*_, *l*_*i*_, *w*_*i*_) the true request, we have $a_{i}^{{}^{\prime }}>a_{i},\;d_{i}^{{}^{\prime }}<d_{i},\;w_{i}^{{}^{\prime }}<w_{i}$, and $l_{i}^{{}^{\prime }}>l_{i}$. We are interested in designing a truthful online channel sharing scheme in which for every *v* = (*v*_1_, *v*_2_, …, *v*_*n*_) with *v*_*i*_ = (*a*_*i*_, *d*_*i*_, *l*_*i*_, *w*_*i*_) and every $v_{i}^{{}^{\prime }}=\left(a_{i}^{{}^{\prime }},\;d_{i}^{{}^{\prime }},l_{i}^{{}^{\prime }},w_{i}^{{}^{\prime }}\right)$, we have $q_{i}(v)w_{i}-p_{i}(v)\geq q_{i}(v_{i}^{{}^{\prime }},v_{-i})w_{i}^{{}^{\prime }}-p_{i}\left(v_{i}^{{}^{\prime }},v_{-i}\right)$, where *v*_−*i*_ = (*v*_1_, …, *v*_*i*−1_, *v*_*i*+1_, …, *v*_*n*_), i.e., the profit of user *i* is maximized if and only if it submits the request for the channel access truthfully.

The truthfulness requirement makes the online channel sharing problem described above significantly difficult. Users may be selfish and seek to manipulate their channel access requests to control the channel allocation result. Since the channel access request is time based, a selfish user can not only rig its channel length, satisfaction value, but also its start and end time. This makes the design of a truthful payment rule that is resistant to all possible misreports extremely difficult. A truthful mechanism might suffer severe degradation in efficiency in order to balance the tradeoff between the truthfulness and efficiency.

### Truthful Channel Sharing Scheme

We propose a truthful channel sharing scheme for the coexistence of multiple MBANs. The truthful channel sharing scheme can enforce users to send their true channel access requests by properly defining the channel allocation and payment rules. We now describe our design in detail. We first look at the scenario where channel access length for each request is unit-length, and then we present the solution for the variable length case. We then design the protocol to support the channel sharing methods based on IEEE 802.15.6 architecture. We derive the following definition of the truthfulness property based on the model given above:

***Definition 1*** ((*a*_*i*_, *d*_*i*_, *l*_*i*_, *w*_*i*_)***-Truthful Channel Sharing)***: *Let* *v*_*i*_ = (*a*_*i*_, *d*_*i*_, *l*_*i*_, *w*_*i*_) *represent user* *i’s true request based on the current bandwidth request of its applications. A channel sharing scheme is* (*a*_*i*_, *d*_*i*_, *l*_*i*_, *w*_*i*_)*-truthful if and only if no user* *i* *can improve its profit by submitting a false request* $v_{i}^{{}^{\prime }}=\left(a_{i}^{{}^{\prime }},\;d_{i}^{{}^{\prime }},l_{i}^{{}^{\prime }},w_{i}^{{}^{\prime }}\right)$ *which has* $a_{i}^{{}^{\prime }}>a_{i},\;d_{i}^{{}^{\prime }}<d_{i},\;\;w_{i}^{{}^{\prime }}<w_{i}$, *and* $l_{i}^{{}^{\prime }}>l_{i}$.

The general design guideline to enforce truthfulness is to make the channel access allocation monotonic and to apply critical-value based pricing by charging each winner the minimum bid required to win the auction [6]. The monotonic allocation makes sure that a user can only win the channel access auction by bidding higher than a threshold bid so as to resist bid cheating. We allow a preemption feature to do a tradeoff between users’ profit and the channel efficiency. Now we give a formal definition of monotonic channel allocation:

***Definition 2 (Monotonic Channel Allocation)***: *A channel allocation is monotonic if a request* *v*_*i*_ = (*a*_*i*_, *d*_*i*_, *l*_*i*_, *w*_*i*_) *dominates the request* $v_{i}^{{}^{\prime }}=\left(a_{i}^{{}^{\prime }},\;d_{i}^{{}^{\prime }},l_{i}^{{}^{\prime }},w_{i}^{{}^{\prime }}\right)$ *denoted by* $\;v_{i}≻v_{i}^{{}^{\prime }}$, *i.e.* $a_{i}^{{}^{\prime }}>a_{i},\;d_{i}^{{}^{\prime }}<d_{i},\;w_{i}^{{}^{\prime }}<w_{i}$, *or* $l_{i}^{{}^{\prime }}>l_{i}$, *we have* *q*_*i*_(*v*)≥*q*_*i*_(*v*′), *where* $v_{j}=v_{j}^{{}^{\prime }}$ *for* *j* ≠ *i*, *i.e., if a user wins the auction by submitting request* $v_{i}^{{}^{\prime }}=\left(a_{i}^{{}^{\prime }},\;d_{i}^{{}^{\prime }},l_{i}^{{}^{\prime }},w_{i}^{{}^{\prime }}\right)$, *it will win the auction by submitting request* *v*_*i*_ = (*a*_*i*_, *d*_*i*_, *l*_*i*_, *w*_*i*_) *assuming all other factors remain the same*.

#### Unit-length Channel Access

In this section, we consider the unit-length case where each time a user requests only one frame, i.e., *l*_*i*_ = 1. We can then simplify the channel access request by defining it as *v*_*i*_ = (*a*_*i*_, *d*_*i*_, *w*_*i*_). In the online channel sharing process, each user submits its request for channel accessto the auctioneer whenever it finds that its MBAN requires some bandwidth for access to the communication between its coordinator and the MBAN end devices. This triggers the channel allocation decision process. This decision process commences at the beginning of each superframe wherein new user requests arrive for channel bandwidth, or when a previous winner finishes its usage of the channel. We call this type of a frame *a critical frame* in this paper.

Driven by the monotonic allocation methodology above, we apply the following channel allocation rule *q* (HIGHEST BID FIRST) to allocate frames to users: for each critical frame *t*, we allocate frame *t* to user *i* with the pending request that satifies the following requirement: *w*_*i*_ = max{*w*_*j*_ : *a*_*j*_ ≤ *t* ≤ *d*_*j*_}, i.e., the highest bid user for the current frame will be served first. We now show that allocation rule *q* has the monotonicity property as below:

**Theorem 1** *The HIGHEST BID FIRST channel allocation scheme q is monotonic, i.e.,* *q*_*i*_(*v*) ≥ *q*_*i*_(*v*′) *if* $v_{i}≻v_{i}^{{}^{\prime }}$.

PROOF. Assume a user *i* loses the auction when she submits a request *v*_*i*_ = (*a*_*i*_, *d*_*i*_, *w*_*i*_). Now instead of *v*_*i*_ = (*a*_*i*_, *d*_*i*_, *w*_*i*_), she tries a false request by $v_{i}^{{}^{\prime }}=\left(a_{i}^{{}^{\prime }},\;d_{i}^{{}^{\prime }},w_{i}^{{}^{\prime }}\right)$, where $v_{i}≻v_{i}^{{}^{\prime }}$. Since $a_{i}^{{}^{\prime }}>a_{i},\;d_{i}^{{}^{\prime }}<d_{i}$ and $\;w_{i}^{{}^{\prime }}<w_{i}$, we have $q_{i}\left(v\right)=q_{i}\left(a_{i},a_{i}^{{}^{\prime }},w_{i}\right)+q_{i}\left(a_{i}^{{}^{\prime }},d_{i}^{{}^{\prime }},w_{i}\right)+q_{i}\left(d_{i}^{{}^{\prime }},d_{i},w_{i}\right)\geq q_{i}\left(a_{i}^{{}^{\prime }},d_{i}^{{}^{\prime }},w_{i}\right)\geq q_{i}\left(a_{i}^{{}^{\prime }},d_{i}^{{}^{\prime }},w_{i}^{{}^{\prime }}\right)=q_{i}\left(v_{i}^{{}^{\prime }}\right).$

For the truthful property to be implementable, the pricing scheme has to be bid independent and monotonic. Otherwise, users can increase their profit by cheating. In online channel auction, a user’s price, when winning the auction, depends on other pending users and their bids. Since the set of pending users depends on time, we need to design a pricing scheme that can remove the time dependency and still be monotonic. This means the price charged when a user cheats is no less than that when it reports the same truthfully. Taking the above into account, we define the payment rule *p* as:

$$\begin{array}{c} p_{i}\left(v\right)=q_{i}\left(v\right)w_{i}-\int_{0}^{w_{i}}q_{i}\left(\left(a_{i},d_{i},\;x\right),v_{-i}\right)dx \end{array}$$ (1)

In other words, a user will only have to pay the smallest value it could have reported in order to receive an allocation. The above mentioned payment rule *p* can be equivalently expressed as follows:

$$\begin{array}{c} p_{i}\left(v\right)=\text{min}\left\{w_{i}^{{}^{\prime }}:q_{i}\left(\left(a_{i},d_{i},w_{i}^{{}^{\prime }}\right),v_{-i}\right)=1\right\} \end{array}$$ (2)

The payment rule *p* defined by Eq (2) depends only on *a*_*i*_, *d*_*i*_ and *v*_−*i*_ so it is value independent. It is also monotonic in that it is non-decreasing in *a*_*i*_ and non-increasing in *d*_*i*_. We now show that the HIGHEST BID FIRST allocation rule *q* combined with the pricing rule in Eq (2) constitutes a truthful channel sharing mechanism as defined in theorem 2 below:

**Theorem 2** *Let* *q*: *v*^*n*^ → {0, 1}^*n*^ *be the HIGHEST BID FIRST channel allocation rule above, and p be the payment rule defined by* Eq (1), *then the channel sharing scheme* (*q*, *p*) *is truthful*.

PROOF. Let’s assume the channel sharing scheme (*q*, *p*) is not truthful, i.e., there is a user *i*, a request vector *v* of true types with *v*_*i*_ = (*a*_*i*_, *d*_*i*_, *w*_*i*_) and a non-truthful request $v_{i}^{{}^{\prime }}=\left(a_{i}^{{}^{\prime }},\;d_{i}^{{}^{\prime }},w_{i}^{{}^{\prime }}\right)$, where $v_{i}≻v_{i}^{{}^{\prime }}$, such that the profit of user *i* if it derives from being truthful is strictly greater than the profit if it submits request truthfully, i.e., $q_{i}(v_{i}^{{}^{\prime }},v_{-i})w_{i}^{{}^{\prime }}-p_{i}\left(v_{i}^{{}^{\prime }},v_{-i}\right)>q_{i}(v)w_{i}^{{}^{\prime }}-p_{i}\left(v\right)$. The pricing scheme defined in Eq (1), can be written as follows:

$$\begin{array}{c} \int_{0}^{w_{i}^{{}^{\prime }}}q_{i}\left(\left(a_{i}^{{}^{\prime }},d_{i}^{{}^{\prime }},x\right),x\right),v_{-i})dx>\int_{0}^{w_{i}}q_{i}\left(\left(a_{i},d_{i},x\right),v_{-i}\right)dx \end{array}$$ (3)

Since we have $w_{i}^{{}^{\prime }}<w_{i}$ and the monotonicity property of allocation *q* in THEOREM 1, we have $\int_{0}^{w_{i}^{{}^{\prime }}}q_{i}\left(\left(a_{i}^{{}^{\prime }},d_{i}^{{}^{\prime }},x\right),x\right),v_{-i})dx>\int_{0}^{w_{i}}q_{i}((a_{i},d_{i},x),v_{-i})dx$, which contradicts Eq (3). This contradiction establishes the truthfulness of the mechanism (*q*, *p*).

Based on the discussion above, we now propose the overall unit-length truthful channel sharing algorithm *κ*. Each user *i* has a trust parameter *α* which is updated each time a frame is allocated to the user. If the user *i* is allocated to the current frame *t*, its trust will be updated by $\alpha_{i}=\frac{m_{i}^{-}-\gamma p_{i}}{M_{i}}$ where $m_{i}^{-}$ is user *i*’s money left before the allocation, γ is the *aggressive factor* which determines how rapidly its trust will decrease as a function of its current money left, and *M*_*i*_ is the money that user *i* is allocated by the central authority. The penalty factor λ is a parameter to decide whether a high bid request can preempt the current low bid request but with unfinished pre-allocated frames. A high penalty factor λ means that the auctioneer will assign a higher priority to the current winner. Algorithm 1 provides the steps to share of channels of unit length in detail.

**Algorithm 1** Unit Length Truthful Online Channel Sharing *κ*

**Input**: A set of requests *v* from *n* users for current frame *t*; current payment state *m*_*i*_ for user *i*.

**Output**: user *k* that is allocated to the current frame *t*

1: *k* = *ϕ*;

2: list = SortBidInNonInceasingOrder(*n*, *t*);

3: list = DeleteExpiredRequest(*n*, *t*);

4: **for** *i* = *HeadOfRequestList*(*list*) **do**

5:  $p_{i}(v)=\text{min}\left\{w_{i}^{{}^{\prime }}:q_{i}((a_{i},d_{i},w_{i}^{{}^{\prime }}),v_{-i})=1\right\}$;

6:  **if** *m*_*i*_ − *p*_*i*_ ≥ 0 **then**

7:   *k* = *i*;

8:   break;

9:  **end if**

10: **end for**

11: **for** *i* = *HeadOfRequestList*(*list*) **do**

12:  $m_{i}=m_{i}^{-}-\gamma p_{i}$

13:  **if** *i* = *k* **then**

14:   $\alpha_{i}=\frac{m_{i}-\gamma p_{i}}{M_{i}}$;

15:  **else**

16:   $\alpha_{i}=\frac{m_{i}+\left(\gamma p_{i}\right)/n}{M_{i}}$;

17:  **end if**

18: **end for**

#### Variable-length Channel Access

In this section, we consider the scenario where users submit requests with a variable number of frames each time. As a result, a request now can be expressed as *v*_*i*_ = (*a*_*i*_, *d*_*i*_, *l*_*i*_, *w*_*i*_), where *l*_*i*_ is the number of frames that user *i* requests. User *i* will gain value *w*_*i*_ if its request is met, i.e., it is allocated at least *l*_*i*_ number of frames between (*a*_*i*_, *b*_*i*_). Note that the number of frames *l*_*i*_ do not have to be continues.

The allocation *q* needs to be monotonic as well, i.e., given the start time and end time of user *i*, the higher the user *i* bids, the higher likehood that user *i* wins. To achieve monotonicity, we apply the same allocation policy as that in the unit length case, i.e., for each frame the auctioneer chooses the qualified user with the highest bid as the winner. It is obvious that if there is no new request coming, the current winner *i* will win the auction repeatedly until it uses up all of its allocated *l*_*i*_ frames and leaves the auction. This means allocation decision occurs only when a winner finishes its channel usage and releases the currently occupied channel, or when a new request arrives.

Since the allocated frames do not have to be contiguous and preemption can increase the overall auction profit, we allow preemption, i.e., if the newly arrived request has a higher bid, we may consider to allocate the current frame to it, so that the overall profit can be improved. This however introduces the risk that the current winner may not be able to obtain its requested frames. This implies that in the end this user will get zero profit although it has been allocated some frames already. Considering this negative effect of preemption, the auctioneer should preempt a winner only if the newly arrived user offers a significantly higher bid.

To prevent unbeneficial preemption, we introduce a bid adjustment procedure to control the preemption frequency by artificially raising the ongoing winner’s bid to raise its priority. For a winning user *i* with request *v*_*i*_ = (*a*_*i*_, *d*_*i*_, *l*_*i*_, *w*_*i*_) and the current frame *t*, suppose user *i* has totally been allocated $l_{i}^{-}$ frames since the start time *a*_*i*_, then we will treat user *i*’s bid at time *t* as: $w_{i}^{t}=\frac{w_{i}}{l_{i}}\lambda^{\varphi_{i}}\geq w_{i}$ where $\varphi_{i}=l_{i}^{-}/l_{i}$ indicates the current state of allocation. Since we would like to raise the priority of user *i* when it is close to getting the total number of requested frames, we let λ ≥ 1, which will determine the auctioneer’s preemption aggressiveness. When λ = 1, it becomes a conventional preemption case. By increasing λ, the auctioneer will assign a higher priority to the current winner since it will add more bids to the winner to protect it from being preempted by the newly arrived user. When λ → ∞, $w_{i}^{t}=\infty$, then the winner will never be preempted until its current request is met. In this case, there is no preemption.

We charge the users who have gained partial access to the requested channel as well. The pricing for user *i* takes place after the allocation, i.e., when *t* > *d*_*i*_. Let $p_{i}^{t}$ denote the price for winning frame *t*. We apply the same policy as in the unit length case to price each frame, i.e., for frame *t* we charge user $p_{i}^{t}\left(v\right)=\text{min}\left\{w_{i}^{{}^{\prime }}:q_{i}^{t}\left(\left(a_{i},d_{i},l_{i},w_{i}^{{}^{\prime }}\right),v_{-i}^{t}\right)=1\right\}$, where $v_{-i}^{t}=(\ldots ,\left(a_{i-1},d_{i-1},l_{i-1},w_{i-1}^{t}\right),\;\left(a_{i+1},d_{i+1},l_{i+1},w_{i+1}^{t}\right),\ldots ,$ $\left(a_{n},d_{n},l_{n},w_{n}^{t})\;\right)$.

Let $S_{i}^{d}$ denote the set of the frames allocated to user *i*, $l_{i}^{d}$ be the number of the allocated frames after end time *d*_*i*_. We call *p*_*i*_ the minimum per-frame price, then we have $p_{i}=\min_{t\in S_{i}^{d}}p_{i}^{t}\left(v\right)$. We charge user *i* by $p_{i}l_{i}^{d}$ so as to remove the time dependency. This is because under the assumption of no early arrival and no late departure, we have $\left(a_{i}^{{}^{\prime }},\;d_{i}^{{}^{\prime }}\right)\subset \left(a_{i},d_{i}\right)$, so that $\min_{t\in {S^{{}^{\prime }}}_{i}^{d}}p_{i}^{t}\left(v\right)>\min_{t\in S_{i}^{d}}p_{i}^{t}\left(v\right)$, where ${S^{{}^{\prime }}}_{i}^{d}$ is the set of frames allocated to user *i* with request $v_{i}^{{}^{\prime }}=\left(a_{i}^{{}^{\prime }},\;d_{i}^{{}^{\prime }},l_{i}^{{}^{\prime }},w_{i}^{{}^{\prime }}\right)$. This implies that the price charge to user *i* is higher when it cheats.

To resist users’ selfish tendency of reporting a larger number of frames than what is required, we introduce a request reject scheme, for a newly arrived request *v*_*i*_ = (*a*_*i*_, *d*_*i*_, *l*_*i*_, *w*_*i*_). If $\frac{l_{i}}{d_{i}-w_{i}}>\frac{\beta_{i}}{N}$, then the request will be rejected, where *N* is the number of users and *β*_*i*_ decides how user *i*’s request can be dependant on user *i*’s financial state indicated by *m*_*i*_. For example *β*_*i*_ can be calculated as: $\beta_{i}=\frac{m_{i}}{\sum_{j\in N}m_{j}}$.

Now we summarize the channel sharing scheme described above as follows. We call a frame *t* critical frame if a newly arrived request starts at frame *t*, or a previous allocation completes at frame *t* − 1. For a new request, if $\frac{l_{i}}{d_{i}-w_{i}}>\frac{\beta_{i}}{N}$, it will be rejected. For the current frame *t*, we calculate user *i*’s frame bid by $w_{i}^{t}=\frac{w_{i}}{l_{i}}\lambda^{\varphi_{i}}$, and then apply the HIGHEST BID FIRST allocation policy to allocate the frame to user *i* with the highest bid $w_{i}^{t}$ that still has enough money to pay for the usage of this frame. For each user *i* that has been allocated $S_{i}^{d}$ number of frames from the request *v*_*i*_ = (*a*_*i*_, *d*_*i*_, *l*_*i*_, *w*_*i*_), we apply the minimum per-frame price scheme, i.e., $p_{i}^{t}=\min_{t\in S_{i}^{d}}p_{i}^{t}\left(v\right)$ to charge it for each frame allocated, so that the overall price user *i* has to pay is $p_{i}^{t}L_{i}$.

Algorithm 2 lists the detailed steps of channel sharing for variable length case. It consists of allocation and pricing algorithms as depicted above together with the related protocols.

**Algorithm 2** Variable Length Truthful Online Channel Sharing *ω*

**Input**: A set of requests *v* from *n* users for *l*_*i*_; current payment state *m*_*i*_ for user *i*.

**Output**: user *k* that is allocated to the current frame *t*

1: *k* = *ϕ*;

2: list = DeleteExpiredRequest(*n*, *t*);

3: **for** *i* = *HeadOfRequestList*(*list*) **do**

4:  **if** user *i* is a new user and $\frac{l_{i}}{d_{i}-w_{i}}>\frac{\beta_{i}}{N}$ **then**

5:   DeleteRequestFromList(list, *i*);

6:  **end if**

7:  calculate user’s bid for frame *t* by $w_{i}=w_{i}^{t}=\frac{w_{i}}{l_{i}}\lambda^{\varphi_{i}}$;

8: **end for**

9: SortBidInNonInceasingOrder(*list*, *t*);

10: **for** *i* = *HeadOfRequestList*(*list*) **do**

11:  $p_{i}^{t}=\text{min}\left\{w_{i}^{{}^{\prime }}:q_{i}\left(\left(a_{i},d_{i},w_{i}^{{}^{\prime }}\right),v_{-i}\right)=1\right\}$;

12:  **if** *m*_*i*_ > 0 **then**

13:   *k* = *i*;

14:   break;

15:  **end if**

16: **end for**

17: **for** *i* = *HeadOfRequestList*(*list*) **do**

18:  **if** if user *i* has been allocated *l*_*i*_ frames **then**

19:   $p_{i}^{t}=\min_{t\in S_{i}^{d}}p_{i}^{t}\left(v\right)$

20:   $p_{i}=p_{i}^{t}*l_{i}$

21:  **end if**

22:  $m_{i}=m_{i}^{-}-\gamma p_{i}$

23:  **if** *i* = *k* **then**

24:   $\alpha_{i}=\frac{m_{i}-\gamma p_{i}}{M_{i}}$;

25:  **else**

26:   $\alpha_{i}=\frac{m_{i}+\left(\gamma p_{i}\right)/n}{M_{i}}$;

27:  **end if**

28: **end for**

We now prove that the channel sharing scheme for variable length described above is truthful by means of the following theorem:

**Theorem 3** *The channel sharing scheme for the variable length case is* (*a*, *d*, *l*, *w*)*-truthful, i.e., no user i can improve its profit by submitting a manipulated request* $v_{i}^{{}^{\prime }}=\left(a_{i}^{{}^{\prime }},\;d_{i}^{{}^{\prime }},l_{i}^{{}^{\prime }},w_{i}^{{}^{\prime }}\right)$ *with* $a_{i}^{{}^{\prime }}>a_{i},\;d_{i}^{{}^{\prime }}<d_{i},\;w_{i}^{{}^{\prime }}<w_{i}$, *and* $l_{i}^{{}^{\prime }}>l_{i}$.

PROOF. Suppose that there is a user *i*, a vector *v* of true request with *v*_*i*_ = (*a*_*i*_, *d*_*i*_, *l*_*i*_, *w*_*i*_), and a manipulated non-truthful request $v_{i}^{{}^{\prime }}=\left(a_{i}^{{}^{\prime }},\;d_{i}^{{}^{\prime }},l_{i}^{{}^{\prime }},w_{i}^{{}^{\prime }}\right)$, where $a_{i}^{{}^{\prime }}>a_{i},\;d_{i}^{{}^{\prime }}<d_{i},\;w_{i}^{{}^{\prime }}<w_{i}$, and $l_{i}^{{}^{\prime }}>l_{i}$. First since the channel sharing scheme removes requests with $\frac{l_{i}}{d_{i}-w_{i}}>\frac{\beta_{i}}{N}$, where *β*_*i*_ and *N* are unknown to user *i*, no user can benefit by lying $l_{i}^{{}^{\prime }}>l_{i}$. We now show that user *i* cannot benefit from rigging its bid *w*_*i*_. For frame *t*, Theorem 1 ensures that there exists a critical value such that user *i* wins only if it bids no less than this value. The proof of truthfulness is similar to that in the unit length case. We do not discuss the details of the proof.

We now consider the arrival time and end time. The monotonicity of the allocation rule makes sure that if user *i* wins with request $v_{i}^{{}^{\prime }}=\left(a_{i}^{{}^{\prime }},\;d_{i}^{{}^{\prime }},l_{i},\;w_{i}\right)$, it wins with *v*_*i*_ = (*a*_*i*_, *d*_*i*_, *l*_*i*_, *w*_*i*_). The minimum price $p_{i}^{{}^{\prime }}=\min_{t\in S_{i}^{{}^{\prime }}}p_{i}^{t}\left(v_{i}^{{}^{\prime }}\right)$ that user *i* needs to pay for winning the auction within $\left(a_{i}^{{}^{\prime }},\;d_{i}^{{}^{\prime }}\right)$ is less than $p_{i}=\min_{t\in S_{i}}p_{i}^{t}\left(v\right)$ with (*a*_*i*_, *d*_*i*_). Since a user *i* who loses does not affect the allocation result, we have $L_{i}^{{}^{\prime }}\leq L_{i}$. Then we have $u_{i}=w_{i}-p_{i}L_{i}\geq u_{i}^{{}^{\prime }}=w_{i}^{{}^{\prime }}-p_{i}^{{}^{\prime }}L_{i}^{{}^{\prime }}$, which means that user *i* cannot improve its profit by submitting a manipulated request $v_{i}^{{}^{\prime }}=\left(a_{i}^{{}^{\prime }},\;d_{i}^{{}^{\prime }},l_{i}^{{}^{\prime }},w_{i}^{{}^{\prime }}\right)$ with $a_{i}^{{}^{\prime }}>a_{i},\;d_{i}^{{}^{\prime }}<d_{i},\;w_{i}^{{}^{\prime }}<w_{i}$, and $l_{i}^{{}^{\prime }}>l_{i}$. Based on the above, we prove that the channel sharing scheme above is indeed truthful in its implementation.

### Truthful On Demand Self Coexistence Protocol

Self coexistence is critical for the safety of MBAN. Driven by the above channel allocation and pricing methods, we now design the Truthful On Demand Self Coexistence(TODEC) protocol in detail based on the framework of IEEE 802.15.6 standard. TODEC is used to achieve efficient and adaptive self coexistence among overlapping IEEE 802.15.6 MBANs.

**Fig 1:**
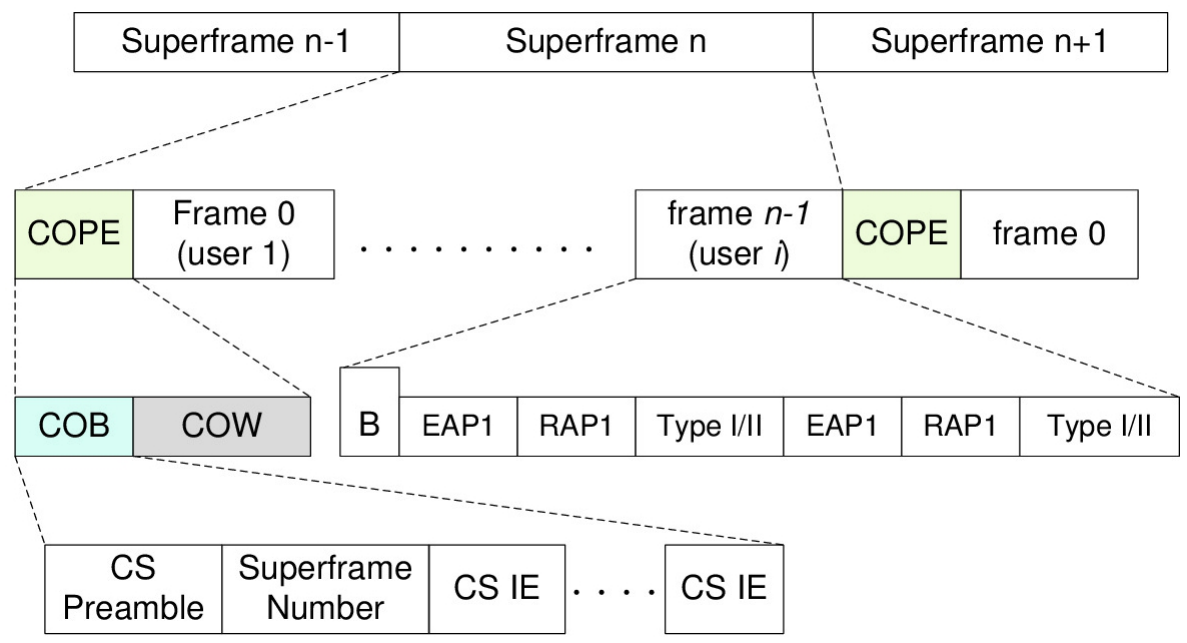
Superframe Structure of a self CO-existence Period (COPE) Protocol. In addition to fields of the conventional frame, the superframe defines a COPE field which consists of coexistence beacon (COB) and coexistence window (COW).

The superframe structure in the self coexistence mode is shown in Fig 1. In addition to the fields of the conventional frame, TODEC defines a self coexistence period (COPE), which consists of coexistence beacon (COB) and coexistence window (COW). During the COB and COW period, no communication within MBAN should happen which is indicated by the conventional beacon (B). The coordinator of the central authority MBAN (CA_MBAN) broadcasts COB to notify other coordinators of MBANs that it works in the self coexistence mode and is ready to take other MBANs requests to share the channel. COB also provides the synchronization information for all MBANs in the channel sharing scenario through CS Preamble, Superframe Number and other CS IE fields. COW period is based on CSMA/CA which allows coordinators of MBANs to send joint requests to CA_MBAN coordinator, the channel request, i.e., *v*_*i*_ = (*a*_*i*_, *d*_*i*_, *l*_*i*_, *w*_*i*_) for MBAN *i*, and channel grant that tells the channel allocation result. Each MBAN in the coexistence mode transmits its allocated number of frames after decoding the broadcast channel grant message during the COW period. The details of the COB, such as CS Preamble and CS IEs are omitted because of space limitations. Note: For the frame definition, please refer to IEEE 802.15.6 standard [1].

**Fig 2:**
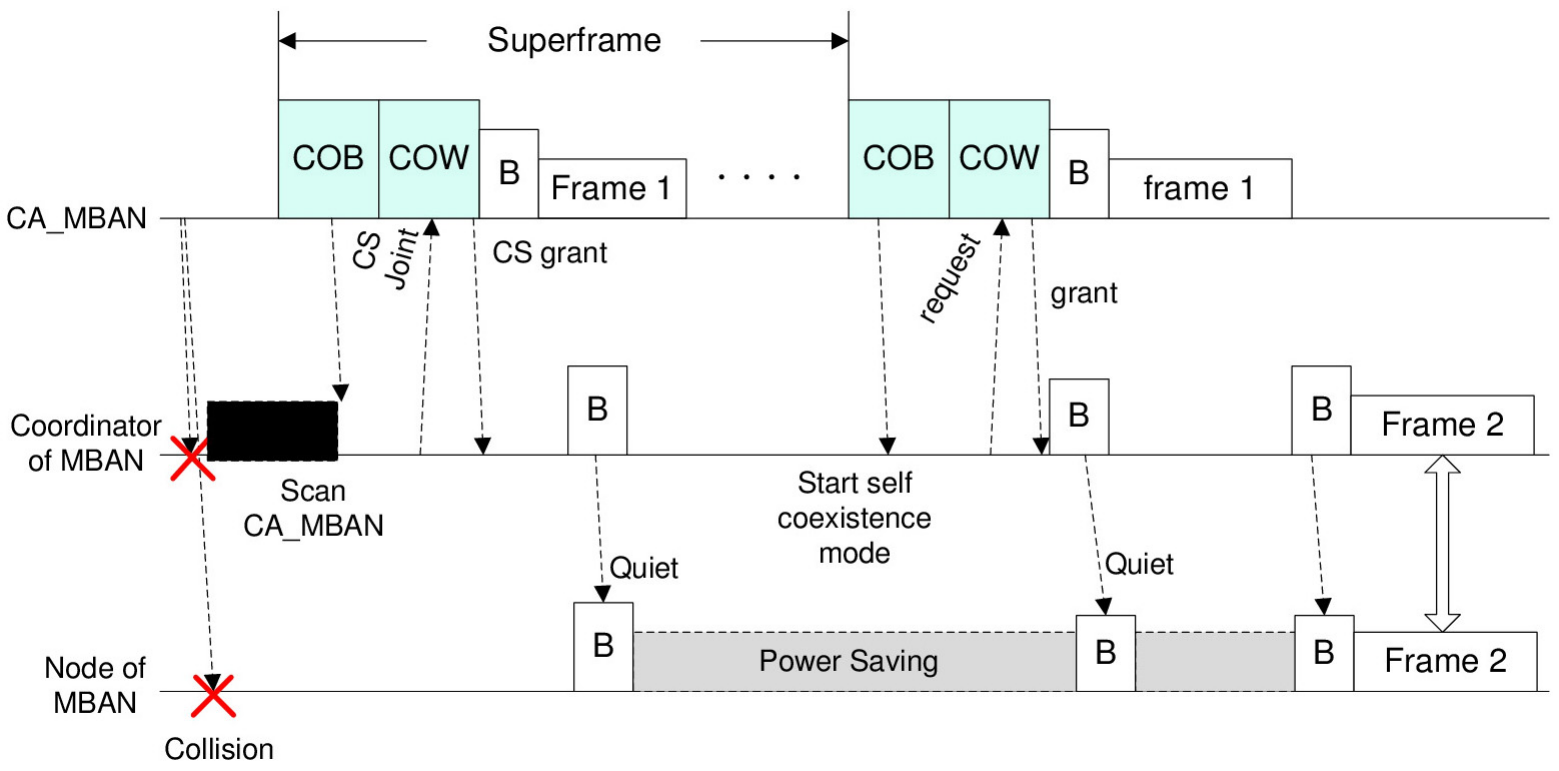
Example of TODEC Process.

A channel sharing based self coexistence mode is trigged by events such as a node or a coordinator detecting severe interference based on the received SINR from the medium as shown in Fig 2. In order to participate in the online channel access auction described in the previous sections, each MBAN (user) needs to get synchronized to the auctioneer, i.e., CA_MBAN. When a MBAN decides to enter into the self coexistence mode, its coordinator firstly searches for nearby CA_MBAN by decoding COB. During the COW period, the coordinator can send a CS joint message to the CA_MBAN to join the channel sharing process. Requests will then be sent to the auctioneer during the COW from the coordinator of MBAN to that of CA_MBAN based on CSMA/CA media access scheme. CA_MBAN will run the truthful channel allocation method proposed above to allow multiple MBANs to share the underlying channel on a frame by frame basis. The CA_MBAN will broadcast the channel access grants to users as part of the COB or in the grant message during COW period. This message indicates which frame has been allocated to which user. Fig 2 shows the above process.

There are four types of messages needed to support the channel sharing process: Channel Sharing Joint (CS Joint), Channel Sharing grant (CS Grant), Channel Request and Grant as shown in Fig 2. CS Joint message is transmitted by the MBAN coordinator to CA_MBAN coordinator to initiate the channel sharing process. CS Grant contains the jointing request acknowledgement information. Grant message contains the confirmation of the channel allocation result. Based on this framework, more flexible schemes may be added, for example, a MBAN can be granted periodical frames and it can ask for more frames or cancel previously allocated frames. The details are omitted here because of space limitations.

The channel allocation decision takes place after the COW period where users submit requests to the auctioneer. The allocation decision algorithm will be trigged only if there is at least one new request arrival or a winner finishes its channel usage and releases the channel. When joining the channel sharing process, user *i* will be assigned a trust parameter by the auctioneer. The auctioneer does this based on the overall QoS requirements of the concerned MBAN and its explanation is beyond the scope of this paper. Each time the allocation algorithm first deletes the expired requests according to the timing information, i.e., *a*_*i*_ and *d*_*i*_, it will apply the HIGHEST BID FIRST allocation policy to allocate the next frame to user *i* with the highest bid that still has a high trust to pay for the usage of the this frame, i.e., *m*_*i*_ − *p*_*i*_ ≥ 0.

### Complexity Analysis

We now analyze time and space complexity of our proposed online channel sharing algorithm. As is known, time complexity is a function describing the amount of time an algorithm takes while space complexity describes the amount of memory (space) an algorithm takes, in terms of the amount of inputs to the algorithm. In our algorithm, the amount of inputs is the number of users *n* for the unit length channel sharing scheme and the number of users *n* and the number of frames Δ each user requests for the variable length channel sharing scheme.

The main component of the unit length channel sharing scheme, as described in Algorithm 1, is to find the highest bid user and the second highest bid user. The algorithm consists of one sorting algorithm and two *for* loops. For time complexity analysis of the algorithm, a comparison sorting algorithm requires linearithmic time *O*(*n* log *n*), and the number of repeated times of each loop is *O*(*n*). In the first *for* loop, a variable, *v*_−*i*_, in the *min*{.} function has (*n* − 1) elements; then, the complexity of this *min*{.} function is *O*(*n*). So, the complexity of this loop is *O*(*n*^2^). The second loop’s complexity is *O*(*n*). Therefore, the time complexity of Algorithm 1 is *O*(*n* log *n*) + *O*(*n*^2^) + *O*(*n*) = *O*(*n*^2^). For space complexity, the algorithm needs space to save the request list which has *n* elements. Other parts of the algorithm, such as the sorting algorithm and for loops, perform operations on the request list. For each element in the request list, the memory consumed by these operations is constant. The memory consumption increases when there are more users. Hence, its space complexity is linear *O*(*n*).

Similarly complexity analysis on the variable length channel sharing scheme, as described in Algorithm 2, is presented as below. Algorithm 2 has one sorting algorithm as well as three *for* loops. For time complexity analysis, a comparison sorting algorithm is *O*(*n* log *n*) while each loop repeats *O*(*n*) times. The complexity of the first and the third loop is *O*(*n*) and *O*(Δ*n*), respectively, where Δ is the number of requested frames. However, the complexity of the second loop is *O*(*n*^2^) because the *min*{.} function in the loop has operations of *O*(*n*). Therefore, the time complexity of Algorithm 2 is *O*(*n* log *n*) + *O*(Δ*n*) + *O*(*n*^2^) = *O*(*n*^2^). For space complexity, the main part of memory is for saving the request list with *n* elements. Likewise, its space complexity is *O*(*n*).

In the next section, we conduct simulations to study the profit trends. In terms of the overall profit performance, we compare it against the earliest deadline first (EDF) and Weighted Fair Queue (WFQ) algorithms.

## Results

In this section, we discuss the result of extensive simulations to study the performance of our algorithm *ω*, specifically, in terms of the competitive ratio and network profit in typical settings of MBANs. In our simulations, we generate *n* MBANs that are randomly placed in a 4 × 4 square meter area and the coverage of each MBAN is 5 meters. We assume any transmission within 4 meters will conflict with each other if the transmitters request the same channel and the same time frames. We generate random channel requests with random bid values and time requirements for truthful MBANs. For a request (*a*, *d*, *l*, *w*), bid *w* is uniformly distributed in [0, *π*], the time gap between two successive requests is uniformly distributed in [1, *δ*] and duration *d* − *a* is uniformly distributed in [*l*, *φ*]. The duration is uniformly distributed in [1, *ρ*], where selfish users can manipulate *π*, *δ*, *φ* and *ρ*. We take the total service time as 10000 time frames which is large enough compared to the time requirements of each request.

The average load of an MBAN in our simulation is (*K*.*J*)/(*T*.*n*), where *K* is the total number of requests, *J* is the average duration of a request and *n* is the total number of MBANs, thus we have *J* = *ρ*/2 and *T* = 10000 frames in our setting. Considering the typical applications of MBANs, we expect the number of MBANs in a unit square meter area to be around 3 so that the total number of MBANs in a 4 × 4 square meter area is 50.

### Competitive Ratio of Algorithm *ω*

When calculating the competitive radio of algorithm *ω*, various parameters, such as penalty factor λ and aggressive factor γ may affect performnce. To see how these parameters affect performance, we first fix penalty factor λ and study the competitive ratios of *ω* while aggressive factor γ varies. Then we fix the aggressive factor γ and study the competitive ratios of *ω* while penalty factor λ changes.

**Fig 3:**
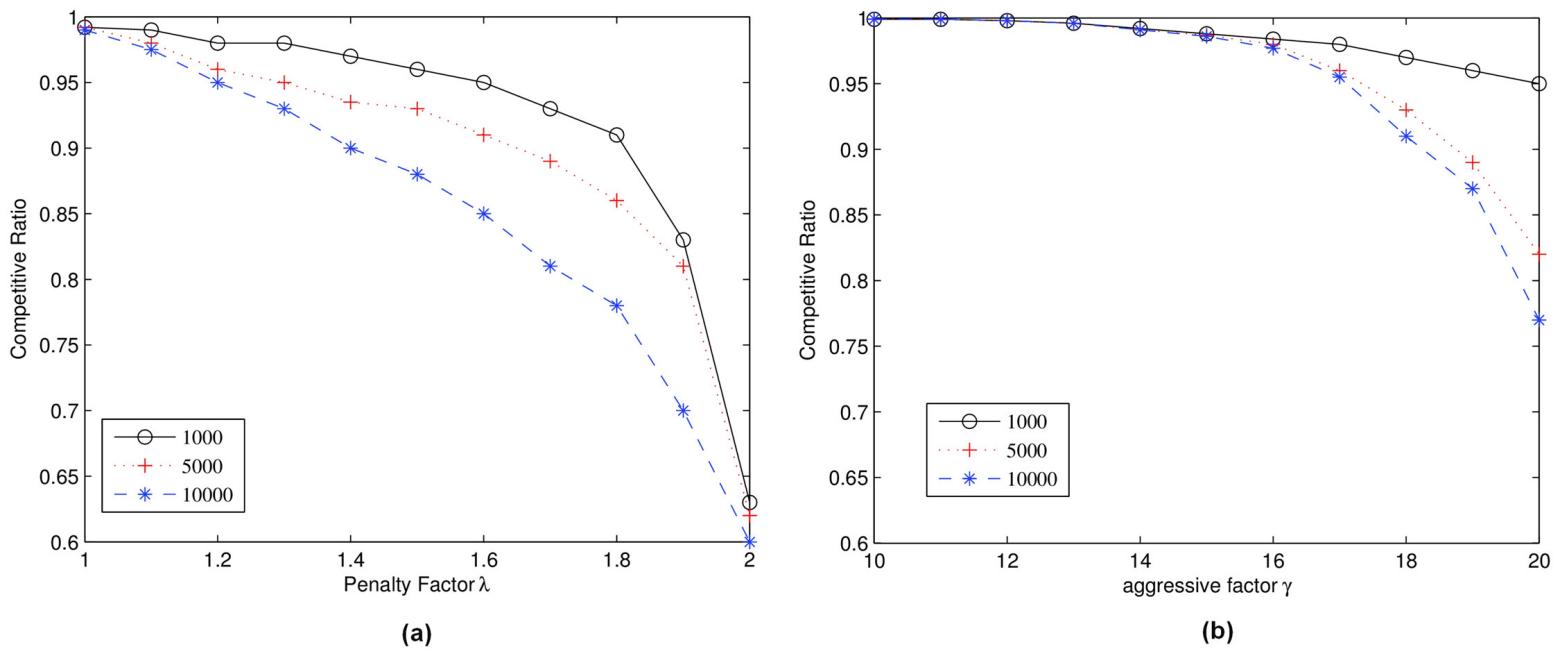
Competitive Ratio. (a):λ = 16; (b):γ = 1.35.

Fig 3(a) shows the competitive ratios of *ω* for various algorithms as a function of penalty factor λ while aggressive factor γ is fixed. Fig 3(b) shows the competitive ratios of algorithm *ω* for various algorithms as a function of aggressive factor γ while penalty factor λ is fixed. By configuring *π*, *δ*, *φ* and *ρ* properly, we can have a different total number of requests. We run 3 cases: 1000 requests, 5000 requests and 10,000 requests. In most cases, algorithm *ω* makes a total profit that is more than 85% of the optimum offline method.

The competitive ratio decreases when penalty factor λ increases. This is because penalty factor λ is a parameter to decide whether a high bid request can preempt the current low bid request but with unfinished pre-allocated frames. A high penalty factor λ means that the auctioneer will assign a higher priority to the current winner since it will add more bids to the winner to protect it from being preempted by the newly arrived user, and as a result, the overall profit decreases. The competitive ratio decreases as aggressive factor γ increases. This is because aggressive factor γ is a parameter that affects how a user’s trust parameter will be updated: a high γ means a user will be punished severely and may lose trust completely, and as a result, it may miss the chance to send its bid because of it being suspended.

The total number of requests also affects the performance of *ω*. We observe that the competitive ratio decreases when the total number of requests increases. This is because *ω* is conservative: the weak preemption in *ω* just tries to satisfy the theoretical bound which may potentially lose some profit. Thus, when there are more requests, the optimal offline algorithm will make a higher profit but the profit made by *ω* will not increase so much.

### Truthfulness of Algorithm *ω*

Truthfulness is the most important characteristic of algorithm *ω*. To study the truthfulness, we introduce selfish users who manipulate their requests by controlling the *π*, *δ*, *φ* and *ρ* parameters to increase their priority. We compare algorithm *ω* to two classic scheduling algorithms based on the earliest deadline first (EDF) and weighted fair queuing (WFQ). EDF always serves user *k* with the earliest deadline, i.e., $k=\arg\;\min_{i\in \{1...n\}}\;\left(d_{i}\right)$, while WFQ picks up the user with the highest bid *l*_*i*_ if all the users are equal, i.e., $k=\arg\;\max_{i\in \{1...n\}}\;\left(l_{i}\right)$. When a request with a larger bid is coming, WFQ will terminate the current assignment with a smaller bid value if necessary. When a request with an earlier deadline is coming, EDF will terminate the current assignment with a smaller bid value if necessary. The competitive ratio and fairness of these two algorithms could be theoretically arbitrarily bad. We conduct simulations to compare them with algorithm *ω* which is theoretically truthful.

**Fig 4:**
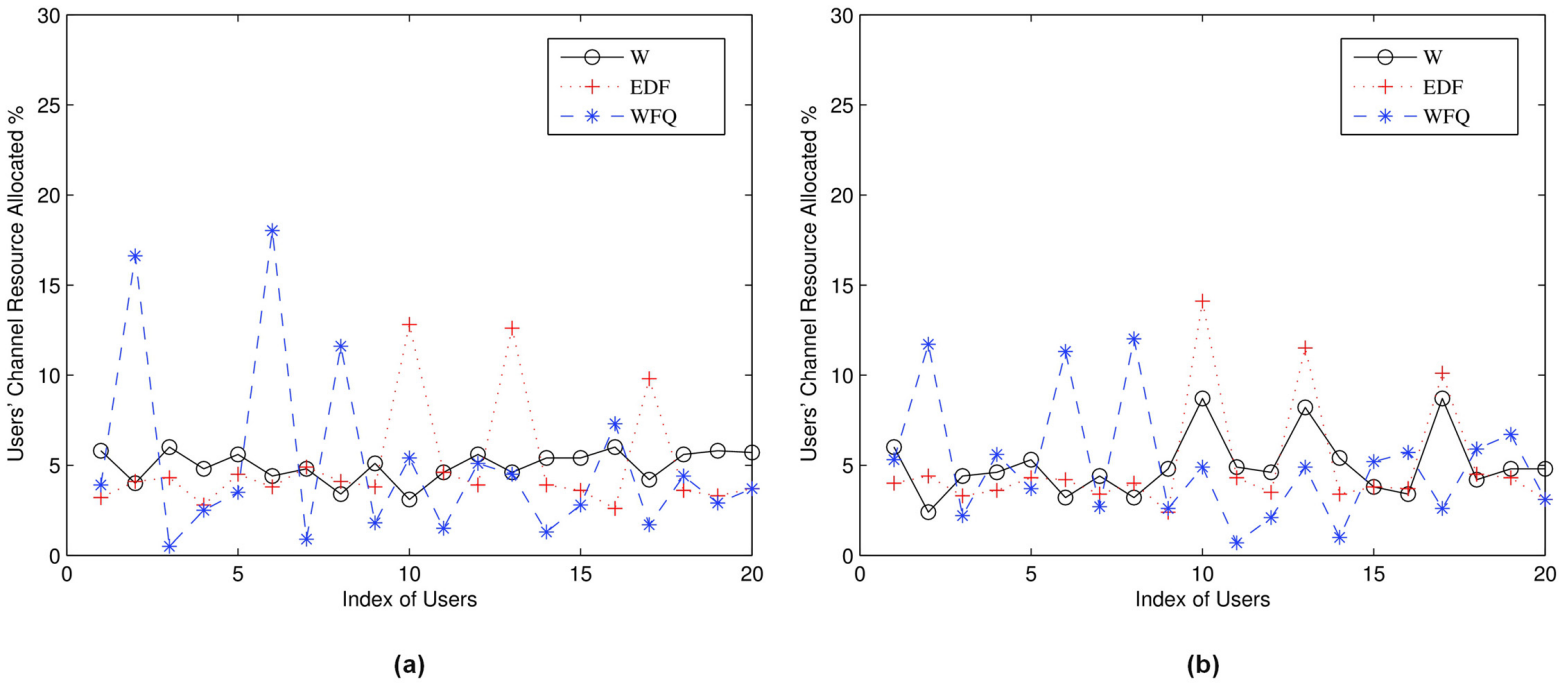
Users’ Channel Allocation vs Selfish Requests. (a):λ = 2.4; (b):λ = 1.2.

**Fig 5:**
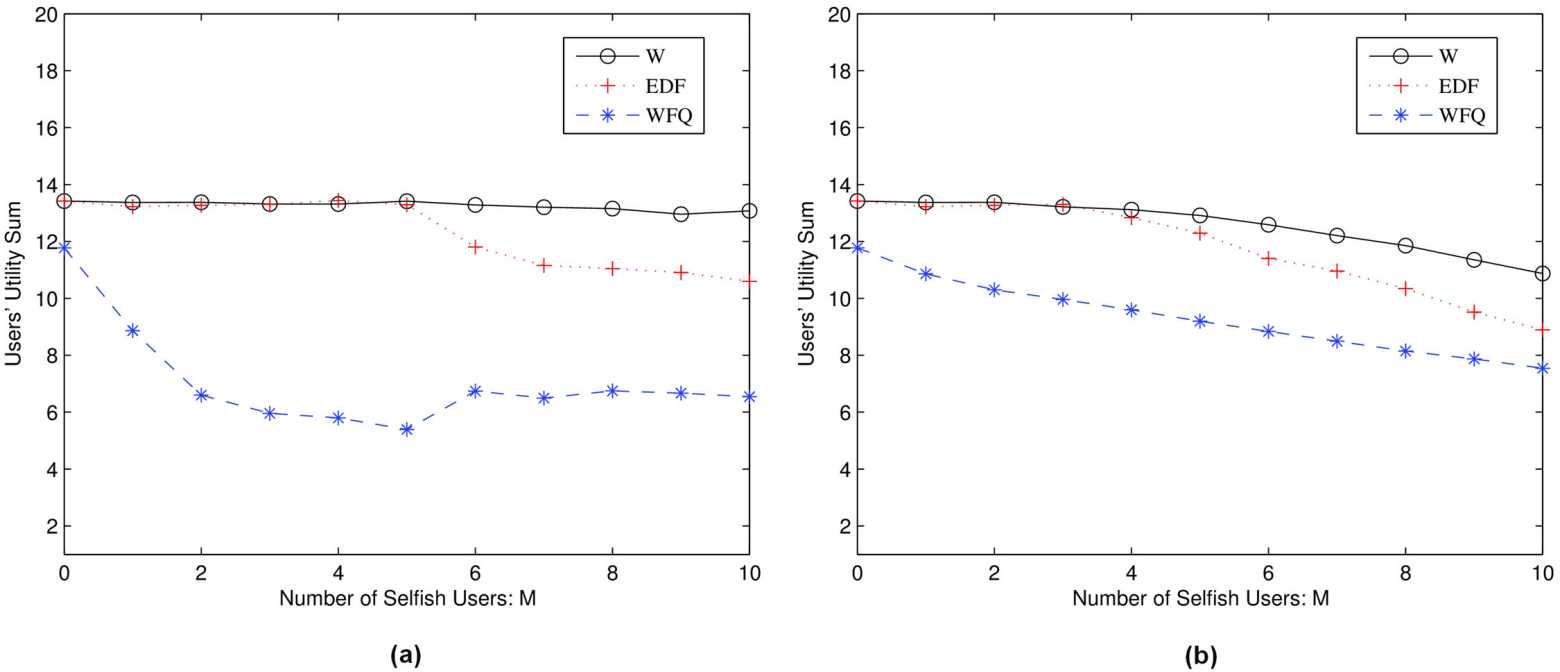
Users’ Overall profit vs Selfish Users. (a): users manipulate *d*_*i*_, *l*_*i*_; (b): users manipulate *w*_*i*_.

**Fig 6:**
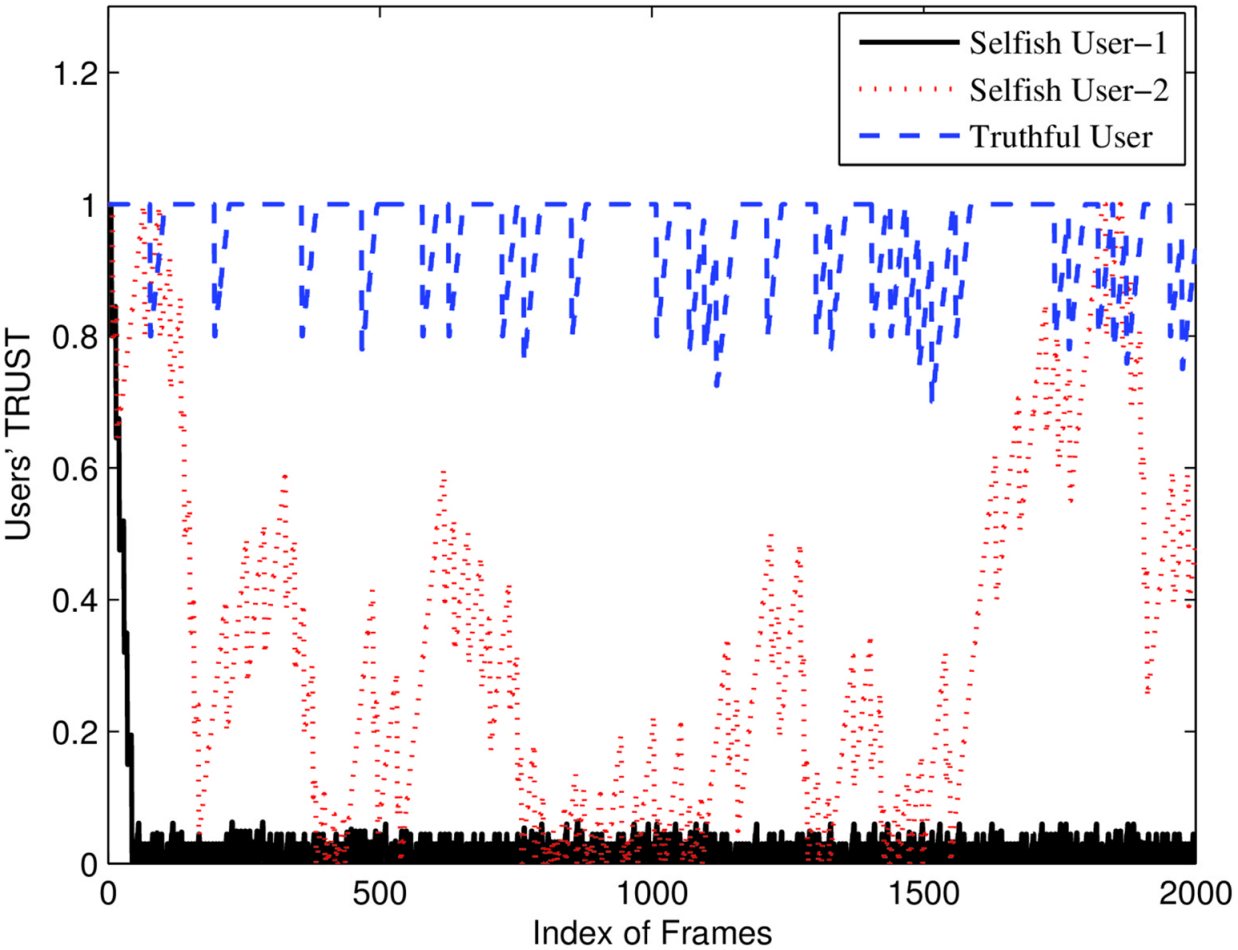
Users’ Trust vs Selfish Users.

In Fig 4(a) and 4(b), we plot the channel allocation result of algorithms *ω*, EDF and WFQ to compare their performance. In Fig 5(a), we define users 2, 8, 10, 13 and 17 as selfish users. Users 2 and 8 manipulate their requests by raising the bids of their requests. Users 10, 13 and 17 decrease the duration of the requests to gain channel resources. EDF and WFQ cannot detect the selfish behaviors of users, so the selfish users benefit from manipulating their requests. Our algorithm *ω*, however, maintains the trust parameter of each user and can detect selfish users and punish them when users’ trust is low. As a result, selfish users 2, 8, 10, 13 and 17 do not obtain extra channel resources but on the contrary obtained lower channel resources than the other truthful users as shown in Fig 6(a). Fig 6(b) shows how penalty factor λ will affect the results.

In Fig 5(a) and 5(b), we plot the overall user profit. Our proposed algorithm *ω* has a high and constant sum of profit when the number of selfish users increases. This is because the selfish users will be detected and some of their requests will be ignored when their trusts are low. The EDF and the WFQ however do not have a trust monitoring scheme so that some selfish users can gain more resources by selfishly manipulating their channel requests, and as a result other honest users will lose channel resources and their overall utilities decrease.

In Fig 6, we plot the users’ trust. We show how the trust parameter of different type of users, i.e,. truthful and selfish users, will behave as a function of time, i.e., as a function of the index of the frame. In Fig 6, we manipulate users’ requests by controlling *π*, *δ*, *φ* and *ρ*, so that users 1 and 2 are selfish users and user 1 is more offensive than user 2. User 1 basically decreases duration and increases bids dramatically in order to have a higher priority. User 2 however employs a try-and-stop policy, i.e., each time it tries to be selfish, it will monitor if it is benefiting. If it believes it is suffering, it will try to be truthful. As a result, from Fig 6, we can see User 1’s trust is almost zero all the time, i.e., it has a bad reputation and will not be allocated any resource. User 2 is randomly trying to manipulate its request parameters and its trust is quite random as well. The truthful user has a good reputation and has a trust value of more than 0.8 in this setting.

## Discussion

In this paper, we have studied online channel sharing problem for medical body area networks (MBANs) where a set of MBANs will bid for leasing a channel for a certain time duration in different locations. We model the channel sharing problem as an online auction for channel access. Our proposed scheme combines a monotonic channel allocation rule and a payment mechanism. We have proposed truthful channel sharing methods for both the unit length and variable length cases. Based on the methods, we designed a flexible and practical self coexistence protocol based on IEEE 802.15.6 standard for MBANs. For a number of variants, we have experimentally shown that the competitive ratios of our methods are within small constant factors of the optimum. Our algorithm results in a profit that is almost optimum and can maintain fairness by detecting and punishing selfish MBANs. Our extensive simulation results show that our scheme can achieve a total profit that is close to that of the offline optimum method in the typical MBAN settings. Truthfulness of our scheme helps to achieve fair channel sharing when there are selfish users, as a result it can achieve a higher total profit than that of other algorithms.

We now discuss related work on allocating channels and highlight similarities as well differences between our work and the existing works. Efficient and truthful mechanisms for various dynamic spectrum assignment problems were proposed [7, 8], as well as double spectrum auctions with provable performance [9, 10]. All these proposals are based on offline models. For the online models, spectrum allocation and truthful schemes in situations where secondary users could bid arbitrarily were proposed [11, 12].

In our work, we use an online model which is similar to the online job scheduling problems [13] to solve channel sharing among MBANs. Various online scheduling problems focus on optimizing different objective functions. The most common objective function is makespan, which is the length of the schedule. Suppose that we are given m identical machines, jobs arrive one by one and no preemption is allowed for this scenario. A number of results have been reported in the literature to improve the upper bounds [6, 10, 14] and lower bounds [15]. Closing the gap between the best lower bound [15] and the upper bound [6] is an open problem. The case where preemption is allowed without penalty has also been investigated [16].

When jobs have deadlines, however, it is usually impossible to finish all the jobs. Thus, another model aims to maximize the profit or the number of completed jobs. There are different variants: preemption-restart, preemption-resume, and preemption-discard. An algorithm was proposed [17], matching the lower bound given in [2]. An online model of maximizing the profit of finished jobs was studied [18] where there is some relationship between the weight and length of a job. A 4-competitive algorithm was proposed for the tight deadline case, and a matching lower bound was given [7]. In this algorithm, it is assumed that preemption is allowed while no penalties will be charged. It was proven that a suitable choice of channel sharing and incentive schemes can reduce collusion incentives among participating nodes [12]. A channel selection algorithm was proposed that optimally selects a channel by minimizing queuing delay [19]. A channel allocation scheme was combined with the routing framework with a view to maximizing the profit in the network [20]. The scheme proposed is decentralized and is restricted to cognitive mesh topologies. A 2 way bargaining scheme was proposed to force selfish SUs to cooperate with PUs so as to guarantee the PU’s QoS requirement [21]. The scheme goes further by offering a mechanism to coordinate the available spectrum among SUs. However, none of the studies discussed above consider the ‘truthfulness’ issue in channel sharing.

A model for spectrum sharing based on truthful mechanisms and virtual currency was proposed [22]. The model is dynamic in which SUs access opportunistically those channels not temporarily used by PUs. It considers a particular case in which every SU estimates capacity of a free channel according to its local information, and sends the valuation (i.e., estimated capacity) to the centralized spectrum manager. This model is somewhat similar to our model, however, the proposed credit based mechanism restricts truthfulness guarantees to a single run where a ‘run’ represents a spectrum usage opportunity. Several recent studies [23–26] investigated various mechanisms of evolutionary game theory to explain the cooperation among selfish individuals, e.g. the prisoner’s dilemma game model and the public goods game model.

A power control algorithm that uses the Signal to interference ratio (SIR) as a cost function has been proposed [27]. The proposed algorithm achieves optimal exploitation of the spectrum with least amount on interference. It achieves fairness by enabling all users to meet their SIR constraints without the need to transmit at high power levels. However, this scheme lacks a pricing scheme to manage interference. A scheme which is suitable for 802.11 wireless LANs that operate in unsupervised contention mode was proposed [28]. Upon winning a channel, this scheme enables the winning node to dynamically adjust its transmission opportunity (in which a node can transmit multiple frames without releasing the channel) leading to better bandwidth utilization and QoS provisioning. This scheme is based on the principles of game theory. Although this scheme improves overall network performance, this scheme does not explain how to bid for a channel successfully.

Truthful spectrum bidding was proposed based on the principles of auction theory [29]. The proposal presents an online version of truthful double auctions for spectrum allocation where the requests arrive in an online fashion. The limitation of this scheme is that it does not facilitate pre-empting existing spectrum usage. A neighbor centric approach for coordinating spectrum sharing among secondary users was proposed [30]. Unlike our approach, wherein all bidders are competing against each other, this approach restricts competing bidders (SUs) of a node to be its immediate neighbors. By considering only the neighbors, it allows for spatial reuse since if a primary user (PU) is using a channel then this channel becomes unavailable for a SU that happens to be its neighbor. Other bidders not in the immediate vicinity of this PU remain unaffected. Extending this concept a bit further, the number of channels won by a bidder in this scheme is a function of the cumulative demand of this bidding node’s conflicting neighbors. If there are a several competing bidders for a set of channels then this becomes a case of ‘excessive demand’ and the auction process proceeds to subsequent rounds with price being incremented at each round. Furthermore, each channel that is bid has some sort of a preference number. The approach makes available for selection only that channel that has the least preference number of the winning bidder’s neighbors, thereby reducing the overall number of bidders. However, unlike our approach, it does not examine specific issues such as the overlapping channel allocation scenario and allocating channels of variable length.

In our future work, we will be dealing with the selection of the central authority without the infrastructure, and the interference from other legacy wireless systems working at the same frequency as MBANs. Our model can also be potentially used in many other application areas, where dynamic spectrum sharing is required, such as cognitive radio networks, wireless industrial control systems, TV white space spectrum management, bandwidth allocation management for cellular phone and vehicular networks and other futuristic mobile broadband systems.

## References

[1] WG802 15—Wireless Personal Area Network (WPAN) Working Group. 802.15.6-2012 - IEEE Standard for Local and metropolitan area networks—Part 15.6: Wireless Body Area Networks. IEEE; 2012. Available from: https://standards.ieee.org/findstds/standard/802.15.6-2012.html [cited 2015 21 December].

[2] Zhou X, Gandhi S, Suri S, Zheng H. eBay in the sky: strategy-proof wireless spectrum auctions. In: Proceedings of the 14th ACM International Conference on Mobile Computing and Networking (MobiCom’08). ACM; 2008. p. 2–13.

[3] Li XY, Wang Y. Simple approximation algorithms and PTASs for various problems in wireless ad hoc networks. Journal of Parallel and Distributed Computing. 2006;66(4):515–530.

[4] Vickrey W. Counterspeculation, auctions, and competitive sealed tenders. The Journal of Finance. 1961;16(1):8–37. 10.1111/j.1540-6261.1961.tb02789.x

[5] Clarke EH. Multipart pricing of public goods. Public Choice. 1971;11(1):17–33. 10.1007/BF01726210

[6] Lavi R, Mu’alem A, Nisan N. Towards a characterization of truthful combinatorial auctions. In: Proceedings of the 44th Annual IEEE Symposium on Foundations of Computer Science. IEEE; 2003. p. 574–583.

[7] Kasbekar G, Proutiere A. Opportunistic medium access in multi-channel wireless systems: a learning approach. In: Proceedings of the 48th Annual Allerton Conference on Communication, Control, and Computing. IEEE; 2010. p. 1288–1294.

[8] Li XY, Xu P, Tang S, Chu X. Spectrum bidding in wireless networks and related. In: Proceedings of the 14th Annual International Conference on Computing and Combinatorics (COCOON 2008). vol. 5092 of Lecture Notes in Computer Science. Springer Berlin Heidelberg; 2008. p. 558–567.

[9] Zhou X, Zheng H. TRUST: a general framework for truthful double spectrum auctions. In: Proceedings of the 28th IEEE International Conference on Computer Communications (INFOCOM 2009). IEEE; 2009. p. 999–1007.

[10] Xu P, Li XY. Online market driven spectrum scheduling and auction. In: Proceedings of the 2009 ACM Workshop on Cognitive Radio Networks (CoRoNet’09). ACM; 2009. p. 49–54.

[11] Xu P, Li XY. TOFU: semi-truthful online frequency allocation mechanism for Wireless Networks. IEEE/ACM Transactions on Networking. 2011;19(2):433–446. 10.1109/TNET.2010.2067223

[12] Sodagari S, Attar A, Bilén SG. On a truthful mechanism for expiring spectrum sharing in cognitive radio networks. IEEE Journal on Selected Areas in Communications. 2011;29(4):856–865. 10.1109/JSAC.2011.110416

[13] Goemans MX, Wein JM, Williamson DP. A 1.47-approximation algorithm for a preemptive single-machine scheduling problem. Operations Research Letters. 2000;26(4):149–154.

[14] Rudin JF III, Chandrasekaran R. Improved bounds for the online scheduling problem. SIAM Journal on Computing. 2003;32(3):717–735. 10.1137/S0097539702403438

[15] Hoogeveen H, Skutella M, Woeginger GJ. Preemptive scheduling with rejection. In: Proceedings of the 8th Annual European Symposium on Algorithms (ESA 2000). vol. 1879 of Lecture Notes in Computer Science. Springer Berlin Heidelberg; 2000. p. 268–277.

[16] Fleischer R, Wahl M. On-line scheduling revisited. Journal of Scheduling. 2000;3(6):343–353. 10.1002/1099-1425(200011/12)3:6<343::AID-JOS54>3.3.CO;2-U

[17] Koren G, Shasha D. Dover: an optimal on-Line scheduling algorithm for overloaded uniprocessor real-time systems. SIAM Journal on Computing. 1995;24(2):318–339. 10.1137/S0097539792236882

[18] Hoogeveen H, Potts CN, Woeginger GJ. On-line scheduling on a single machine: maximizing the number of early jobs. Operations Research Letters. 2000;27(5):193–197.

[19] Do CT, Tran NH, Hong CS, Lee S, Lee JJ, Lee W. A lightweight algorithm for probability-based spectrum decision scheme in multiple channels cognitive radio networks. IEEE Communications Letters. 2013;17(3):509–512. 10.1109/LCOMM.2013.012313.122589

[20] Amini RM, Dziong Z. An economic framework for routing and channel allocation in cognitive wireless mesh networks. IEEE Transactions on Network and Service Management. 2014;11(2):188–203. 10.1109/TNSM.2013.120413.120533

[21] Zhang B, Kai C, Yu JL, Cheng S. Two-level bargaining game modeling for cooperation stimulation in spectrum leasing. IEICE Transactions on Communications. 2013;E96.B(7):1953–1961.

[22] Vidal JR, Pla V, Guijarro L, Martinez-Bauset J. Dynamic spectrum sharing in cognitive radio networks using truthful mechanisms and virtual currency. Ad Hoc Networks. 2013;11(6):1858–1873.

[23] Wang J, Xia C, Wang Y, Ding S, Sun J. Spatial prisoner’s dilemma games with increasing size of the interaction neighborhood on regular lattices. Chinese Science Bulletin. 2012;57(7):724–728. 10.1007/s11434-011-4890-4

[24] Zhu CJ, Sun SW, Wang L, Ding S, Wang J, Xia C. Promotion of cooperation due to diversity of players in the spatial public goods game with increasing neighborhood size. Physica A: Statistical Mechanics and its Applications. 2014;406:145–154.

[25] Wang L, Xia C, Wang J. Coevolution of network structure and cooperation in the public goods game. Physica Scripta. 2013;87(5):055001 10.1088/0031-8949/87/05/055001

[26] Xia C, Miao Q, Wang J, Ding S. Evolution of cooperation in the traveler’s dilemma game on two coupled lattices. Applied Mathematics and Computation. 2014;246:389–398.

[27] Al-Gumaei YA, Noordin KA, Reza AW, Dimyati K. A new SIR-based sigmoid power control game in cognitive radio networks. PLoS ONE. 2014;9(10):e109077 10.1371/journal.pone.0109077 25286044 PMC4186786

[28] Ghazvini M, Movahedinia N, Jamshidi K. GTXOP: a game theoretic approach for QoS provisioning using transmission opportunity tuning. PLoS ONE. 2013;8(5):e62925 10.1371/journal.pone.0062925 23650539 PMC3641108

[29] Wang S, Xu P, Xu X, Tang S, Li X, Liu X. TODA: truthful online double auction for spectrum allocation in wireless networks. In: Proceedings of the 2010 IEEE Symposium on New Frontiers in Dynamic Spectrum. IEEE; 2010. p. 1–10.

[30] Manickam S, Marina MK, Pediaditaki S, Nekovee M. An iterative and truthful multi-unit auction scheme for coordinated sharing of spectrum white spaces. SIGMETRICS Performance Evaluation Review. 2014;42(3):8–11. 10.1145/2695533.2695537

